# 2,5-Dimethyl-1,3-dinitro­benzene

**DOI:** 10.1107/S1600536811031424

**Published:** 2011-08-11

**Authors:** Dean H. Johnston, Heather M. Crather

**Affiliations:** a Department of Chemistry, Otterbein University, Westerville, OH 43081, USA

## Abstract

The title compound, C_8_H_8_N_2_O_4_, was prepared *via* the nitration of *p*-xylene. The mol­ecules are stacked along the *c* axis in an antiparallel manner. The two nitro groups are rotated relative to the benzene ring with dihedral angles of 44.50 (7) and 31.67 (8)°. The tilt of the nitro groups allows the formation of C—H⋯O inter­actions between the ring C—H and nitro groups of adjacent mol­ecules creating puckered sheets perpendicular to the *c* axis. The H atoms of the methyl group in the 5-position are disordered (60° rotation) with an occupancy of 0.616 (19) for the major component. The crystal was found to be a non-merohedral twin with a twin law [−1 −0.002 0.005, 0.00031 −1 0.002, 0.118 −0.007 1] corresponding to a rotation of 180° about the reciprocal axis (001) and refined to give a minor component fraction of 0.320 (2).

## Related literature

For the synthesis and properties of dinitro derivatives of *p*-xylene, see: Kobe & Hudson (1950 ▶); Johnson & Northcott (1967 ▶); Liu *et al.* (2005*a* ▶). For single-crystal diffraction studies of dinitro­toluene, see: McCrone (1954 ▶); Nie *et al.* (2001 ▶); Hanson *et al.* (2004 ▶). For single-crystal diffraction studies of nitro derivatives of simple aromatic compounds, see: Ori *et al.* (1989 ▶); Graham *et al.* (2004 ▶); Liu *et al.* (2005*b* ▶); Demartin *et al.* (2004 ▶). For discussions of non-conventional hydrogen bonding in nitro­aromatics and other compounds, see: Desiraju (2005 ▶); Gagnon *et al.* (2007 ▶).
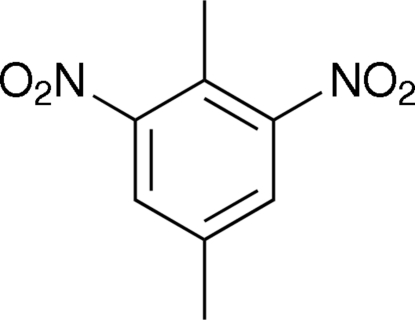


## Experimental

### 

#### Crystal data

C_8_H_8_N_2_O_4_
*M* *_r_* = 196.16
Monoclinic, 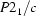

*a* = 12.582 (3) Å
*b* = 9.3868 (17) Å
*c* = 7.3565 (14) Å
β = 91.963 (6)°
*V* = 868.3 (3) Å^3^
*Z* = 4
Mo *K*α radiation
μ = 0.12 mm^−1^
*T* = 200 K
0.40 × 0.40 × 0.30 mm

#### Data collection

Bruker SMART X2S benchtop diffractometer
Absorption correction: multi-scan (*TWINABS*; Bruker, 2009 ▶) *T*_min_ = 0.76, *T*_max_ = 0.96
2868 measured reflections
1540 independent reflections
1307 reflections with *I* > 2σ(*I*)
*R*_int_ = 0.043

#### Refinement

*R*[*F* ^2^ > 2σ(*F* ^2^)] = 0.039
*wR*(*F* ^2^) = 0.103
*S* = 1.04
1540 reflections
132 parameters
H-atom parameters constrained
Δρ_max_ = 0.23 e Å^−3^
Δρ_min_ = −0.15 e Å^−3^

### 

Data collection: *APEX2* and *GIS* (Bruker, 2009 ▶); cell refinement: *SAINT* (Bruker, 2009 ▶); data reduction: *SAINT*; program(s) used to solve structure: *SHELXS97* (Sheldrick, 2008 ▶); program(s) used to refine structure: *SHELXL97* (Sheldrick, 2008 ▶) and *OLEX2* (Dolomanov *et al.*, 2009 ▶); molecular graphics: *PLATON* (Spek, 2009 ▶), *Mercury* (Macrae *et al.*, 2008 ▶) and *POV-RAY* (Cason, 2004 ▶); software used to prepare material for publication: *publCIF* (Westrip, 2010 ▶).

## Supplementary Material

Crystal structure: contains datablock(s) I, global. DOI: 10.1107/S1600536811031424/zl2394sup1.cif

Structure factors: contains datablock(s) I. DOI: 10.1107/S1600536811031424/zl2394Isup2.hkl

Supplementary material file. DOI: 10.1107/S1600536811031424/zl2394Isup3.mol

Supplementary material file. DOI: 10.1107/S1600536811031424/zl2394Isup4.cml

Additional supplementary materials:  crystallographic information; 3D view; checkCIF report

## Figures and Tables

**Table 1:** Hydrogen-bond geometry (Å, °)

*D*—H⋯*A*	*D*—H	H⋯*A*	*D*⋯*A*	*D*—H⋯*A*
C3—H3⋯O1^i^	0.95	2.41	3.340 (2)	165
C5—H5⋯O3^ii^	0.95	2.47	3.207 (2)	134

Symmetry codes: (i) 
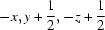
; (ii) 
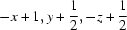
.

**Fig. 1.**
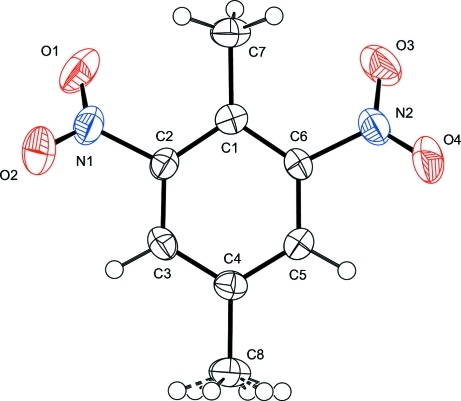
The molecular structure of the title compound drawn with 50% probability displacement ellipsoids for non-H atoms and showing the atom labeling scheme.

**Fig. 2.**
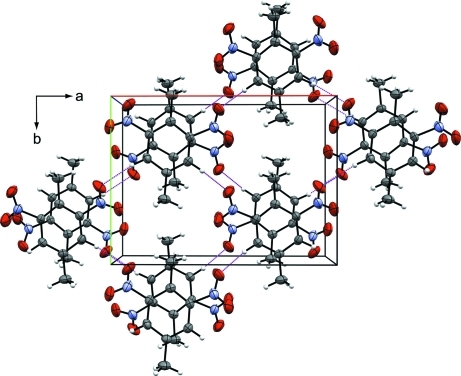
The packing of the title compound viewed along the c axis. Interactions between oxygen atoms and H atoms on the aromatic ring are highlighted with dashed lines. The minor component of the disordered methyl group is not shown.

**Fig. 3.**
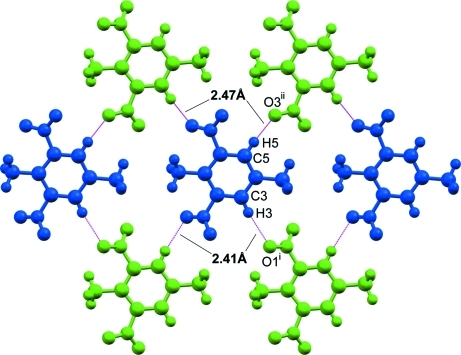
A view down the c axis of one layer formed by C—H···O interactions between hydrogen atoms on the aromatic ring and the nitro groups on adjacent molecules. The different colored molecules are related by a twofold screw axis. The minor component of the disordered methyl group is not shown. Additional donor/acceptor distances and angles are listed in Table 1.

**Fig. 4.**
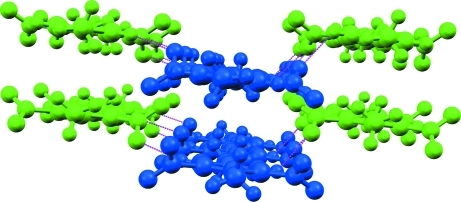
A view approximately down the b axis highlighting the C—H···O interactions between adjacent molecules. The color scheme is the same as that used for Figure 3. The minor component of the disordered methyl group is not shown.

## supplementary crystallographic information

### Comment

Nitro-derivatives of *para*-xylene have been prepared as synthetic intermediates and as energetic materials. There are three possible isomers for the dinitro derivative of *p*-xylene, with studies showing that the major product is the 2,3-dinitro isomer, with exact amounts dependent on reaction conditions (Kobe & Hudson, 1950, Johnson & Northcott, 1967).

The title compound was prepared as a solid derivative of *para*-xylene for a qualitative organic analysis laboratory course. The intended product was the mono-nitro derivative, but it appears the major product was the dinitro product. Large (~1 cm) needle crystals were obtained by vapor diffusion of *n*-pentane into a diethyl ether solution of the compound. The lower solubility of the 1,3-dinitro product relative to the other isomers (Kobe & Hudson, 1950) likely favored formation of crystals of the single isomer.

The dihedral angles between the plane of the benzene ring (Fig. 1) and the two nitro groups are 44.50 (7) and 31.67 (8)°, within the range observed for similar methyl-substituted nitrobenzene derivatives (Demartin, *et al.*, 2004, Liu, *et al.* 2005*a*). The molecules are packed along the *c* axis (Fig. 2) with the rings nearly parallel to each other with an interplane angle of 0.63 (2) ° and interplane spacings (centroid to plane) of 3.648 Å and 3.659 Å. The positioning of the nitro groups enables the formation of non-conventional hydrogen bonds (Desiraju, 2005) between the aromatic C—H and nitro group oxygen atoms of adjacent molecules as illustrated in Figures 3 and 4 (for measurements see Table 1). This type of C—H···O interaction is often found in the structures of simple nitroarenes (Gagnon *et al.*, 2007). These interactions combine to create a network of puckered sheets perpendicular the *c* axis.

### Experimental

Concentrated nitric acid (4 ml) and concentrated sulfuric acid (4 ml) were placed in a round-bottom flask equipped with a Claisen adapter, thermometer and condenser. Approximately 4.5 ml of *p*-xylene was slowly added to the nitric/sulfuric acid mixture, ensuring that the internal temperature did not exceed 323–328 K. After the addition was complete, the mixture was heated for an additional 15 min at 323–328 K. The reaction mixture was cooled to room temperature, poured into 40 ml of cold water and cooled to produce the crystals of the crude nitration product.

The product was recrystallized by vapor diffusion of *n*-pentane into a diethyl ether solution. Large translucent needles formed after two weeks. The melting point of this crystal was determined to be 398.4 (1) K by DSC, in agreement with literature values (Johnson & Northcott, 1967, Liu *et al.*, 2005*a*).

A small, optically clear crystal was cut and selected from the larger crystal under a polarizing microscope. Data sets on three separate crystals selected from different parts of the larger crystal all showed significant non-merohedral twinning.

### Refinement

The twin law, [-1 -0.002 0.005 0.00031 -1 0.002 0.118 -0.007 1], corresponding to a rotation of 179.9° about the reciprocal axis (0 0 1) was determined using the *CELL_NOW* program (Bruker AXS, 2009). Integration and absorption correction (TWINABS, Bruker AXS 2009) gave 1050 unique reflections in domain 1, 1012 unique reflections in domain 2, and 1223 unique overlapping reflections, or 37 percent overlapping reflections. The structure was solved using the non-overlapping reflections from both domains (HKLF 4). The structure was refined using corrected reflections from only the major component including overlaps (HKLF 5). Refinement produced a minor twin component fraction of 0.320 (2).

All hydrogen atoms were located in the difference map and refined with the atom positions constrained to appropriate positions with C—H distances of 0.95 Å (aromatic carbon atoms) or 0.98 Å (methyl groups). The methyl group in the 5-position was modeled as an idealized disordered methyl group with hydrogen atoms in two positions rotated 60° from each other. The occupancy for the major methyl group orientation was 0.62 (2). A riding model was used for all H atoms with *U*_iso_(H) = 1.2 times *U*_iso_ (aromatic) or 1.5 times *U*_iso_ (methyl carbon atoms).

### Crystal data

**Table:**

C_8_H_8_N_2_O_4_	*D*_x_ = 1.501 Mg m^−^^3^
*M**_r_* = 196.16	Melting point: 398.4(1) K
Monoclinic, *P*2_1_/*c*	Mo *K*α radiation, λ = 0.71073 Å
*a* = 12.582 (3) Å	Cell parameters from 2569 reflections
*b* = 9.3868 (17) Å	θ = 3.5–24.7°
*c* = 7.3565 (14) Å	µ = 0.12 mm^−^^1^
β = 91.963 (6)°	*T* = 200 K
*V* = 868.3 (3) Å^3^	Block, clear colourless
*Z* = 4	0.40 × 0.40 × 0.30 mm
*F*(000) = 408	

### Data collection

**Table:**

Bruker SMART X2S benchtop diffractometer	1540 independent reflections
Radiation source: fine-focus sealed tube	1307 reflections with *I* > 2σ(*I*)
doubly curved silicon crystal	*R*_int_ = 0.043
Detector resolution: 8.3330 pixels mm^-1^	θ_max_ = 25.1°, θ_min_ = 2.7°
ω scans	*h* = −14→15
Absorption correction: multi-scan (TWINABS; Bruker, 2009)	*k* = 0→11
*T*_min_ = 0.76, *T*_max_ = 0.96	*l* = 0→8
2868 measured reflections	

### Refinement

**Table:**

Refinement on *F*^2^	Primary atom site location: structure-invariant direct methods
Least-squares matrix: full	Secondary atom site location: difference Fourier map
*R*[*F*^2^ > 2σ(*F*^2^)] = 0.039	Hydrogen site location: difference Fourier map
*wR*(*F*^2^) = 0.103	H-atom parameters constrained
*S* = 1.04	*w* = 1/[σ^2^(*F*_o_^2^) + (0.0667*P*)^2^ + 0.0091*P*] where *P* = (*F*_o_^2^ + 2*F*_c_^2^)/3
1540 reflections	(Δ/σ)_max_ < 0.001
132 parameters	Δρ_max_ = 0.23 e Å^−^^3^
0 restraints	Δρ_min_ = −0.15 e Å^−^^3^

### Special details

**Table:**

Geometry. All e.s.d.'s (except the e.s.d. in the dihedral angle between two l.s. planes) are estimated using the full covariance matrix. The cell e.s.d.'s are taken into account individually in the estimation of e.s.d.'s in distances, angles and torsion angles; correlations between e.s.d.'s in cell parameters are only used when they are defined by crystal symmetry. An approximate (isotropic) treatment of cell e.s.d.'s is used for estimating e.s.d.'s involving l.s. planes.
Refinement. Refinement of *F*^2^ against ALL reflections. The weighted *R*-factor *wR* and goodness of fit *S* are based on *F*^2^, conventional *R*-factors *R* are based on *F*, with *F* set to zero for negative *F*^2^. The threshold expression of *F*^2^ > σ(*F*^2^) is used only for calculating *R*-factors(gt) *etc*. and is not relevant to the choice of reflections for refinement. *R*-factors based on *F*^2^ are statistically about twice as large as those based on *F*, and *R*- factors based on ALL data will be even larger.

### Fractional atomic coordinates and isotropic or equivalent isotropic displacement parameters (Å^2^)

**Table:**

	*x*	*y*	*z*	*U*_iso_*/*U*_eq_	Occ. (<1)
C1	0.26217 (13)	0.14793 (18)	0.1233 (2)	0.0281 (4)	
C2	0.16617 (13)	0.22131 (17)	0.1367 (2)	0.0295 (4)	
C3	0.15567 (13)	0.36737 (19)	0.1375 (2)	0.0323 (4)	
H3	0.0873	0.409	0.1472	0.039*	
C4	0.24416 (13)	0.45384 (18)	0.1243 (2)	0.0304 (4)	
C5	0.34132 (13)	0.38694 (17)	0.1104 (2)	0.0302 (4)	
H5	0.4038	0.4426	0.0995	0.036*	
C6	0.34884 (11)	0.2402 (2)	0.1123 (2)	0.0279 (4)	
C7	0.26878 (16)	−0.01147 (18)	0.1075 (2)	0.0402 (5)	
H7A	0.2846	−0.0527	0.2278	0.06*	
H7B	0.3253	−0.0369	0.0251	0.06*	
H7C	0.2007	−0.0488	0.0594	0.06*	
C8	0.23407 (16)	0.61384 (19)	0.1237 (3)	0.0438 (5)	
H8A	0.1999	0.6449	0.0087	0.066*	0.616 (19)
H8B	0.3049	0.6568	0.1369	0.066*	0.616 (19)
H8C	0.1908	0.6439	0.2252	0.066*	0.616 (19)
H8D	0.2639	0.6521	0.2385	0.066*	0.384 (19)
H8E	0.1589	0.6403	0.1103	0.066*	0.384 (19)
H8F	0.273	0.6532	0.022	0.066*	0.384 (19)
N1	0.06685 (12)	0.14007 (19)	0.1519 (2)	0.0426 (4)	
N2	0.45679 (12)	0.18168 (17)	0.1043 (2)	0.0400 (4)	
O1	0.06653 (12)	0.03920 (18)	0.25790 (19)	0.0589 (5)	
O2	−0.00966 (11)	0.17884 (17)	0.0609 (2)	0.0687 (5)	
O3	0.47671 (12)	0.07044 (16)	0.18286 (19)	0.0563 (4)	
O4	0.52229 (10)	0.2493 (2)	0.0219 (3)	0.0705 (5)	

### Atomic displacement parameters (Å^2^)

**Table:**

	*U*^11^	*U*^22^	*U*^33^	*U*^12^	*U*^13^	*U*^23^
C1	0.0337 (9)	0.0284 (9)	0.0222 (8)	−0.0015 (7)	0.0018 (7)	−0.0006 (7)
C2	0.0270 (8)	0.0312 (10)	0.0305 (9)	−0.0052 (7)	0.0026 (7)	−0.0034 (7)
C3	0.0273 (9)	0.0355 (10)	0.0344 (10)	0.0063 (7)	0.0022 (8)	−0.0045 (8)
C4	0.0352 (9)	0.0270 (9)	0.0292 (8)	0.0021 (7)	0.0029 (7)	−0.0014 (7)
C5	0.0290 (8)	0.0307 (9)	0.0311 (9)	−0.0044 (7)	0.0018 (7)	0.0002 (8)
C6	0.0242 (8)	0.0317 (9)	0.0278 (9)	0.0046 (8)	−0.0004 (7)	−0.0007 (7)
C7	0.0518 (10)	0.0267 (10)	0.0424 (10)	0.0015 (8)	0.0063 (9)	−0.0010 (8)
C8	0.0549 (11)	0.0266 (10)	0.0503 (11)	0.0042 (8)	0.0053 (10)	0.0001 (9)
N1	0.0316 (9)	0.0459 (10)	0.0506 (10)	−0.0103 (7)	0.0078 (8)	−0.0142 (9)
N2	0.0302 (8)	0.0434 (10)	0.0462 (10)	0.0080 (7)	−0.0006 (8)	−0.0023 (8)
O1	0.0573 (9)	0.0583 (10)	0.0621 (9)	−0.0275 (7)	0.0170 (8)	0.0006 (8)
O2	0.0321 (8)	0.0726 (11)	0.1003 (13)	−0.0080 (7)	−0.0140 (9)	−0.0069 (10)
O3	0.0478 (9)	0.0547 (10)	0.0655 (9)	0.0192 (7)	−0.0093 (7)	0.0075 (8)
O4	0.0345 (7)	0.0712 (10)	0.1076 (13)	0.0074 (8)	0.0281 (9)	0.0149 (11)

### Geometric parameters (Å, °)

**Table:**

C1—C2	1.397 (2)	C7—H7B	0.98
C1—C6	1.397 (2)	C7—H7C	0.98
C1—C7	1.503 (2)	C8—H8A	0.98
C2—C3	1.377 (3)	C8—H8B	0.98
C2—N1	1.471 (2)	C8—H8C	0.98
C3—C4	1.384 (2)	C8—H8D	0.98
C3—H3	0.95	C8—H8E	0.98
C4—C5	1.381 (2)	C8—H8F	0.98
C4—C8	1.507 (2)	N1—O2	1.210 (2)
C5—C6	1.380 (2)	N1—O1	1.227 (2)
C5—H5	0.95	N2—O3	1.2154 (19)
C6—N2	1.468 (2)	N2—O4	1.218 (2)
C7—H7A	0.98		
			
C2—C1—C6	112.13 (15)	H8A—C8—H8B	109.5
C2—C1—C7	123.13 (15)	C4—C8—H8C	109.5
C6—C1—C7	124.56 (15)	H8A—C8—H8C	109.5
C3—C2—C1	125.06 (15)	H8B—C8—H8C	109.5
C3—C2—N1	115.70 (15)	C4—C8—H8D	109.5
C1—C2—N1	119.24 (15)	H8A—C8—H8D	141.1
C2—C3—C4	120.39 (15)	H8B—C8—H8D	56.3
C2—C3—H3	119.8	H8C—C8—H8D	56.3
C4—C3—H3	119.8	C4—C8—H8E	109.5
C5—C4—C3	117.05 (15)	H8A—C8—H8E	56.3
C5—C4—C8	121.84 (15)	H8B—C8—H8E	141.1
C3—C4—C8	121.11 (15)	H8C—C8—H8E	56.3
C6—C5—C4	120.89 (15)	H8D—C8—H8E	109.5
C6—C5—H5	119.6	C4—C8—H8F	109.5
C4—C5—H5	119.6	H8A—C8—H8F	56.3
C5—C6—C1	124.46 (15)	H8B—C8—H8F	56.3
C5—C6—N2	115.87 (15)	H8C—C8—H8F	141.1
C1—C6—N2	119.67 (15)	H8D—C8—H8F	109.5
C1—C7—H7A	109.5	H8E—C8—H8F	109.5
C1—C7—H7B	109.5	O2—N1—O1	124.35 (16)
H7A—C7—H7B	109.5	O2—N1—C2	117.61 (17)
C1—C7—H7C	109.5	O1—N1—C2	118.03 (15)
H7A—C7—H7C	109.5	O3—N2—O4	123.52 (15)
H7B—C7—H7C	109.5	O3—N2—C6	118.60 (16)
C4—C8—H8A	109.5	O4—N2—C6	117.87 (15)
C4—C8—H8B	109.5		
			
C6—C1—C2—C3	0.6 (2)	C2—C1—C6—C5	−1.4 (2)
C7—C1—C2—C3	−174.82 (17)	C7—C1—C6—C5	173.92 (17)
C6—C1—C2—N1	−179.20 (14)	C2—C1—C6—N2	177.72 (14)
C7—C1—C2—N1	5.4 (2)	C7—C1—C6—N2	−7.0 (2)
C1—C2—C3—C4	0.1 (3)	C3—C2—N1—O2	44.2 (2)
N1—C2—C3—C4	179.84 (15)	C1—C2—N1—O2	−136.03 (18)
C2—C3—C4—C5	0.0 (2)	C3—C2—N1—O1	−134.69 (17)
C2—C3—C4—C8	179.58 (16)	C1—C2—N1—O1	45.1 (2)
C3—C4—C5—C6	−0.8 (2)	C5—C6—N2—O3	147.42 (16)
C8—C4—C5—C6	179.64 (16)	C1—C6—N2—O3	−31.8 (2)
C4—C5—C6—C1	1.6 (3)	C5—C6—N2—O4	−31.1 (2)
C4—C5—C6—N2	−177.55 (14)	C1—C6—N2—O4	149.73 (18)

### Hydrogen-bond geometry (Å, °)

**Table:**

*D*—H···*A*	*D*—H	H···*A*	*D*···*A*	*D*—H···*A*
C3—H3···O1^i^	0.95	2.41	3.340 (2)	165.
C5—H5···O3^ii^	0.95	2.47	3.207 (2)	134.

Symmetry codes: (i) −*x*, *y*+1/2, −*z*+1/2; (ii) −*x*+1, *y*+1/2, −*z*+1/2.
